# Low intensity pulsed ultrasound information technology intervention in diagnosis and prediction of Muscle Atrophy

**DOI:** 10.12669/pjms.37.6-WIT.4839

**Published:** 2021

**Authors:** Zhijun Sun

**Affiliations:** 1 Zhijun Sun, Master of Degree. Department of Physical Education Teaching, Tianjin University of Commerce, Beichen 300134, Tianjin, China

**Keywords:** LIPUS, information technology, MA, weight-bearing exercise

## Abstract

**Objective:**

To discuss the effects and function of LIPUS on muscle atrophy (MA), analysis from various aspects through the study of low-intensity pulsed ultrasound (LIPUS) information technology intervention (ITI) in diagnosis and the prediction of muscle atrophy..

**Method:**

In this study conducted in our university, 74 healthy female SD rats aged three months, weighing 100-200g were selected. All rats were placed in sterile cages from June 2020 to September 2020. They were divided into three groups. In the OVO group and OVE group, the mice are treated with LIPUS, Finally, the changes of body weight, grasping power, biochemical indexes and glycogen content of gastrocnemius muscle were analyzed and recorded to explore the effect and value of LIPUS ITI combined with intermittent weight-bearing exercise in the treatment of MA

**Results:**

After weight-bearing running, the body weight of model (OVO) group, exercise (OVE) group and NC group had significant statistical significance (P<0.01). It was found that the weight of OVE group was much more as compared to OVO group. There was significant difference in body weight between OVO group and NC group (P<0.05). After LIPUS treatment, it was found that the weight of OVO group, OVE group, LIPUS group and OVE +LIPUS group increased. Compared with the NC group, there was significant statistical difference (P<0.01).

**Conclusion:**

Low intensity pulsed ultrasound ITI has a good effect on improving MA, so as to effectively improve the weight of gastrocnemius muscle. The combined application of the two is better for the improvement of muscular atrophy.

## INTRODUCTION

Muscle atrophy (MA) refers to the disorder of protein metabolism in muscle tissue, the enhancement of proteolysis of muscle, the thinning or even disappearance of muscle fibers. It is often accompanied by decrease of protein synthesis in muscle tissue and the increase of protein degradation.^1^,^2^ In the process of aging, the body will also have MA. Most of the loss of skeletal muscle quality is due to MA caused by the long-term imbalance between muscle protein synthesis and muscle protein decomposition rate.^3^ MA affects people’s health, brings great trouble to people’s daily life.^4^

In this study, the ovariectomized rat model was established. They were divided into different groups. After that, the square algorithm of correlation coefficient of Karl Pearson, root mean square error algorithm and R square algorithm are applied to the model of muscle force prediction. Finally, the changes of body weight, grasping power, biochemical indexes and glycogen content in gastrocnemius muscle were analyzed. This study provides a reliable reference direction and guidance for clinical treatment of MA caused by menopause.

## METHODS

Seventy-four healthy female SD (Sprague Dawley) rats aged three months, and the average weight was 100~200g were selected as the subjects. The study was performed from June 2020 to September 2020. All rats were kept in sterile cages. The national standards for rodents were strictly followed. All animal experiment plans had been approved by Ethical Committee in Tianjin University of Commerce at March 10, 2021.

After one week of adaptive feeding, rats were randomly divided into three groups: control group, ovariectomy operation group and ovariectomy exercise group. 18 rats in the control group were recorded as negative control (NC) group. There were 28 rats in the ovariectomy operation group and the ovariectomy exercise group, which were respectively recorded as OVO group and OVE group.

Rats in OVO group and OVE group were anesthetized. The anesthesia injection used was 30 g/L barbital sodium injection. It was injected into the abdominal cavity of rats to complete anesthesia. Then, rats were placed on the operating table in a prone position for hair removal, skin preparation and disinfection. Then, the subcutaneous tissue and the abdominal muscles were separated. Tweezers were used to remove the surrounding fat and ovaries. Hemostatic forceps were used to ligate the catgut. After that, bilateral ovaries of the rats were removed. Finally, the incision was cleaned and sutured. After anesthetizing the mice in the normal control group, the skin incision was made on both sides of the ventral back, and then the adipose tissue near the ovary was removed. The control group did not need bilateral ovariectomy.

In OVE group, the weight parameters of Bedford TG were the same. Each rat bore 35% of its own weight. First, the rats were fed with adaptive exercise for six weeks, 30 minutes a day, 10 meters per minute. The slope of the running platform was set to 0 and the speed was 20 meters per minute. Exercise was carried out in six groups every day, the exercise time of each group was five minutes, and the interval time was 2 minutes. The total training period of OVE group was 10 weeks. At the end of exercise training, 8 mice in each group were taken out for testing. Then, rats in OVO group were randomly divided into 10 rats in each group, namely OVO group and ultrasound treatment group. The ultrasound treatment group was recorded as LIPUS group. In the same way, the rats in OVE group were randomly divided into two groups, 10 rats in each group, namely OVE group and ovariectomy combined with ultrasound treatment group (OVE +LIPUS group). Then, the LIPUS therapy instrument was used to treat the OVE +LIPUS group and LIPUS group.

### Evaluation algorithm of muscle strength prediction

The square algorithm of correlation coefficient of Karl Pearson, root mean square error algorithm and R square algorithm were applied to the model of muscle force prediction.^5^-^7^ The correlation between muscle force and actual force could be effectively evaluated, and the range of the value was between 1 and-1. If the value was positive, there was a positive correlation between the two variables. If the value was negative, there was a negative correlation between the two variables.


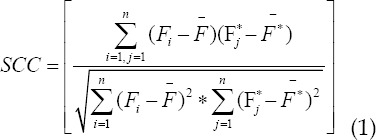


In the above equation, the average value of the actual force was expressed by 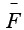. The estimation power and the average of the estimation power were expressed by 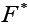 and 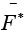 respectively. n was the sample length. If the value was smaller, there was a very low correlation between the actual force and the estimation force, and there was a great difference between them, and the model effect was not superior. The main calculation principle of root mean square error algorithm was as follows:


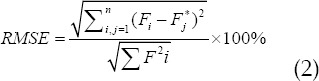


If the calculated value was larger, the estimation effect was the worse, and there was a great difference between the estimated force and the actual force. The smaller the calculated value was, the better was the estimation effect. The difference between the estimated force and the actual force was very small, and the result was almost close.

The main principle of R square algorithm was to describe and explain the change of dependent variable by fitting model. Larger value showed that the fitting model had certain advantages and could describe the variation of independent variables well. If the value was larger, there was a big difference between the predicted muscle force and the actual force.


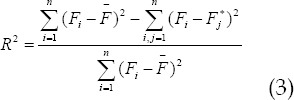


The larger the calculated results, smaller was the difference between the estimated force and the actual force.

## RESULTS

**Fig.1:**
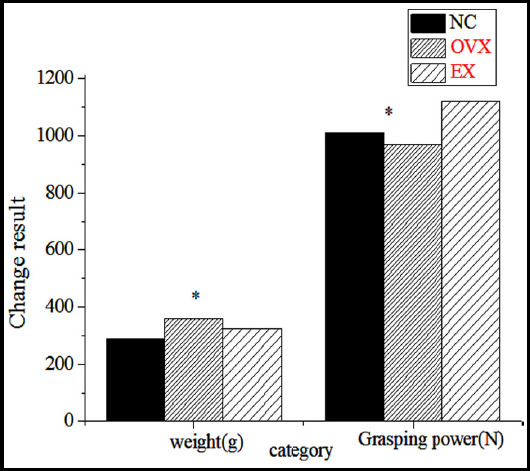
Changes of body weight and grasping power of rats during exercise (* indicates P<0.01).

**Fig.2:**
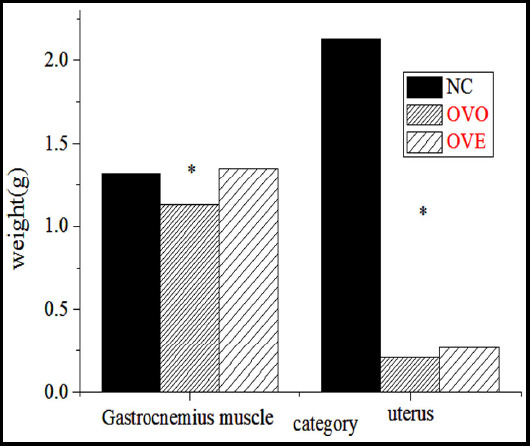
Changes of gastrocnemius muscle weight and uterus weight in rats during exercise (*indicates P<0.01).

The body weight, grasping power, gastrocnemius weight and uterus weight of the rats were calculated after exercise (as can be seen from Fig.1 and Fig.2). The body weight of OVO group, OVE group and NC group had significant statistical significance (P<0.01). The body weight of OVO and OVE rats increased significantly. The body weight of OVO group was compared with that of OVE group. It was found that the body weight of OVE group was relatively light, and there was a very significant statistical significance between the two groups (P<0.01). The rats in OVE group had the largest grasping force, followed by OVO group (P<0.01). It was found that the weight of gastrocnemius muscle in OVE group was the largest and that of gastrocnemius muscle in OVO group was the lightest (P<0.05). The weight of uterus in OVO group and OVE group was lower than that in the NC group, showing statistical difference (P<0.01). Therefore, appropriate intermittent weight-bearing exercise could effectively control the weight, and could effectively increase the weight of gastrocnemius muscle of rats, so as to optimize and improve the skeletal muscle strength of rats, but exercise could not improve the uterus.

After weight-bearing exercise training, the serum calcium content of OVO group and OVE group was not much higher than that of the NC group (P > 0.05). The serum phosphorus content of OVO group and OVE group was significantly higher than that of the NC group (P<0.01). Lactate dehydrogenase (LDH) activity of the three groups was compared. The activity of LDH in OVO group and OVE group was significantly higher than that of the other three groups (P<0.01). The activity of Creatine Kinase (CK) in the three groups were compared. Compared with the NC group, the activity of CK was higher in OVO group, but lower in OVE group (P<0.01). After that, the muscle glycogen content of gastrocnemius was compared among the three groups. Compared with the NC group, the glycogen content of OVO group decreased significantly, but that of OVE group increased (P<0.01).

**Fig. 3:**
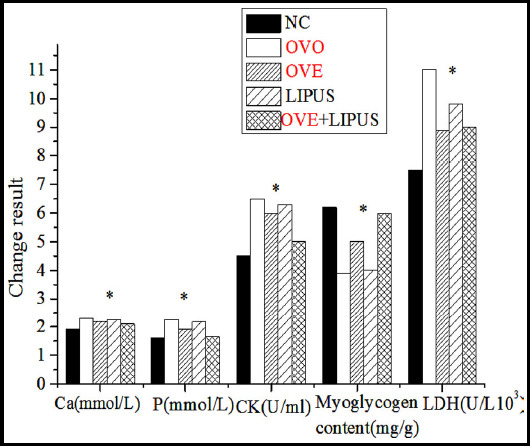
Changes of serum indexes and myoglycogen content (*indicates P<0.01).

As given in Fig.3, the serum index and the content of glycogen in gastrocnemius muscle tissue of rats treated with LIPUS after weight-bearing exercise had changed to some extent. Compared with the control group, the calcium concentration of the other four groups was higher. However, there was no significant difference in calcium concentration between the OVE +LIPUS group and the control group (P > 0.05). Compared with the phosphorus content of the five groups, the phosphorus content of the other four groups was significantly higher than that of the control group (P<0.01). Compared with OVO group, the phosphorus content in OVE group and OVE +LIPUS group was lower than that in OVO group (P<0.01). After that, the CK activity of the five groups were compared, and it was found that the CK activity of the other four groups was significantly higher than that of the control group. Compared with OVO group, the activity of CK in OVE group and OVE +LIPUS group was lower than that in OVO group (P<0.01). As regards LDH activity changes, the relationship between each group and CK activity was the same. The content of glycogen in gastrocnemius muscle was compared among the five groups. Compared with the control group, the glycogen content of the other four groups decreased significantly (P<0.05). Moreover, the muscle glycogen content of OVE group and OVE +LIPUS group was compared with OVO group which showed that the content of muscle glycogen was higher in OVE group and OVE +LIPUS group (P<0.05).

## DISCUSSION

With the increase of age, the ovarian function of women after menopause deteriorates, and the level of endogenous estrogen secretion decreases, which leads to a series of metabolic diseases.^8^ Studies have found that, the postmenopausal women’s skeletal muscle mass and/or function is low, which is mainly manifested as a decrease in the number of skeletal muscles, a decline in skeletal muscle function, and a decline in muscle strength (especially explosive power).^9^-^11^ Low-intensity pulsed ultrasound (LIPUS), as a non-invasive, safe and convenient treatment method, is popular.^12^ Studies have shown that, LIPUS has good therapeutic effects on muscle, tendon, and soft tissue injury, and can promote muscle cell proliferation and protein synthesis.^13^,^14^

In this study, the PILUS technology combined with intermittent weight-bearing exercise was used to treat muscle wasting symptoms in ovariectomized rats. Rats were divided into different groups and treated with different methods. After the ovariectomy of rats, the estrogen secretion in the body decreased which would lead to obesity in rats. However, after weight-bearing exercise training and LIPUS treatment, its weight had been greatly reduced, which showed that weight-bearing running could effectively promote the increase of skeletal muscle tissue fiber content, which had a great role in improving weight. Some researchers have found that the effect of ovariectomy on weight and energy metabolism regulation of rats may be similar to that of high-fat diet. At the same time, it was found that the changes of body weight and energy metabolism caused by ovariectomy and high-fat diet may not directly affect the life span of female rats.^15^-^17^ Other researchers have found that weight-bearing anti resistance exercise could improve skeletal muscle tissue. This study results were consistent with the results of previous studies.^18^-^20^

## CONCLUSIONS

The intervention of LIPUS information technology combined with intermittent weight-bearing exercise can have a good auxiliary treatment effect on the improvement of MA in postmenopausal women. The combination of these two methods has unique advantages. As a new, safe and effective treatment modality, it is worth to be widely used and promoted in clinical practice. However, in this study, only the effects of these two methods on the improvement of MA were analyzed and discussed, and the most fundamental molecular biological mechanism are not explored in detail. In the future research, from the most fundamental point of view, the mechanism of this effect can be studied.
